# Forest City Society of Dental Surgeons

**Published:** 1869-06

**Authors:** Henri L. Ambler

**Affiliations:** Cleveland


HENRI L. AMBLER,
*Recording Secretary.*

### FOREST CITY SOCIETY OF DENTAL SURGEONS.

CLEVELAND, June 1st, 1869.

The first annual meeting of the Forest City Society of Dental Surgeons, was held at the office of Dr. Strickland. The object of this Society is to acquire a better knowledge and development of Dental science and art. To render more successful and complete all operations for preserving and restoring the Dental organism. To elevate members in all that pertains to professional character and practice; bringing them into closer union, for their own benefit and the good of mankind. A proposition for membership must be made in writing, endorsed by two members in good standing. It then lies over until the next meeting, when if they pass the examination of the council, which consists of the officers and three other members, then the candidate, be he not black-balled, pays $5.00, and signs the Constitution.

The following were chosen officers for the ensuing year: President, B. Strickland; Vice-President, B. F. Whitslar; Recording Secretary, H. L. Ambler, Corresponding Sec'y, Chas. Buffett; Treasurer, F. S. Slosson; Councilmen, Drs. Terry, Palmer and Butler.

L. BUFFETT remarked at length upon his successfully treating pericementitis by hypodermic injection of morphia. The matter was made clear by demonstration; he deserves much credit for his untiring experiments in this direction.

H. L. AMBLER exhibited an artificial nose he had made out of rubber, for a patient who had lost all of his nose, but the tip and a portion of the alæ.

Dr. BUTLER remarked at length upon the action of arsenious acid on the tooth structure and dental pulp. He never uses it for obtunding sensitive dentine, as the result is surely pernicious to the well being of the tooth, destroying its vitality either more or less. He says cobalt is the best preparation for a devitalizer of the pulp, and advises its use.

Dr. SPELMAN said the arsenious acid was taken up by the blood, and poisoned the pericementum, causing inflammation.

Dr. PALMER exhibited some drawings and beautiful models of the teeth, giving appropriate names to all the fissures and cusps of the teeth, with proper indications for filling, in order to restore their beauty and strength. He divides the mouth into four sections, and with his demonstrations of "lines of beauty," the matter is made plain, and reduced to a practical and scientific basis, different from anything we have seen or heard of, doing the author great credit.

The delegates to the American Dental Association, appointed by the President, are Drs. Palmer and Butler.

The meeting was one of profit and pleasure, each taking a lively interest in all the proceedings; it being one of the charter principles of this society. that drones will not be tolerated.

